# Radiotherapeutic alternatives for previously irradiated recurrent gliomas

**DOI:** 10.1186/1471-2407-7-167

**Published:** 2007-08-30

**Authors:** Stephanie E Combs, Jürgen Debus, Daniela Schulz-Ertner

**Affiliations:** 1 University Hospital of Heidelberg, Department of Radiation Oncology, Im Neuenheimer Feld 400, 69120 Heidelberg, German

## Abstract

Re-irradiation for recurrent gliomas has been discussed controversially in the past. This was mainly due to only marginal palliation while being associated with a high risk for side effects using conventional radiotherapy.

With modern high-precision radiotherapy re-irradiation has become a more wide-spread, effective and well-tolerated treatment option. Besides external beam radiotherapy, a number of invasive and/or intraoperative radiation techniques have been evaluated in patients with recurrent gliomas.

The present article is a review on the available methods in radiation oncology and summarizes results with respect to outcome and side effects in comparison to clinical results after neurosurgical resection or different chemotherapeutic approaches.

## Background

### "Measure a thousand times – and cut once"

This turkish proverb represents the effort that was put on the issue of re-irradiation in patients with recurrent gliomas. In the past, re-irradiation was thought to be associated with a high incidence of severe treatment-related side effects and was therefore prescribed only reluctantly. A number of reports, however, have suggested that re-irradiation may not be followed by the high incidence of side-effects as feared [1]. Improvement in imaging techniques as well as the establishment of high -precision radiotherapy techniques such as stereotactic radiosurgery (SRS) or fractionated stereotactic radiotherapy (FSRT) in the clinical routine have enabled the radiation oncologist to precisely define a target volume and to describe it with with stereotactic coordinates after targeting with stereotactic methods (*measure a thousand times*) and to deliver high local doses to this area (*and cut once*). Therefore, re-irradiation has become a safe and effective means in controlling recurrent gliomas.

It is known that local radiotherapeutic treatment is a main component in the treatment of astrocytomas after primary diagnosis. For low-grade tumors, althogh the exact time-point of radiotherapy (RT) after primary diagnosis is discussed controversially, most patients are treated at some point during the course of their disease [2,3]. For anaplastic astrocytoma, there is a clear indication for RT after primary diagnosis following neurosurgical resection; presently, combined radiochemotherapy is being evaluated for WHO Grade III tumors (anaplastic astrocytomas, AA). In patients with WHO grade IV astrocytomas, glioblastoma multiforme (GBM), RT is considered the standard postoperative measure, and, if the overall performance status of the patient allows, to be conducted as combined radiochemotherapy with temozolomide [4,5].

Conventional RT is the standard of care radiotherapy approach in patients with progressive gliomas, independently of histology. However, size of the treatment volume varies, from small safety margins of 0.5 to 2 cm for low-grade astrocytomas, to a 2–3 cm safety margin in the treatment volume for anaplastic astrocytomas and high-grade gliomas alike. The total recommended dose amounts to 54 Gy (1.8 Gy qd) for low-grade gliomas, and 60 Gy for GBM, in a fractionation of 5 × 2 Gy/week. Therefore, the majority of patients with recurrent gliomas have been previously exposed to high doses of RT.

Optimization of RT techniques and addition of novel chemotherapeutic substances such as temozolomide have helped significantly increase overall survival for patients with gliomas. Depending on the histologic classification, however, the time to progression may be few months to a number of years, however, tumor progression can be observed in the vast majority of patients. In this situation the neuro-oncologist is faced with a difficult task: Therapy options at the time of recurrence are often limited. Surgery should be considered in all patients, however, gliomas are infiltrative growing tumors and the risk for surgery-associated side effects should be weighed carefully against the possibility and benefit of a surgical intervention [6,7]. Harsh et al. reported median overall survival times of 36 weeks for GBM and 88 weeks for AA after re-operation [8]. A study published by Ammirati et al. corroborated these results with a median survival time of 36 weeks after re-operation [9]. Repeat surgery may not be feasible in a number of patients because of tumor infiltration into eloquent areas of the brain.

Systemic therapies such as chemotherapy have been used widely as single agent or combined treatments, however, often show only modest benefit [10]. Additionally, nowadays, a large group of patients has been exposed to CHT during primary therapy, expecially patients with GBM, potentially limiting the efficacy of chemotherapy or leaving the patients with a decreased bone marrow reserve.

In 1999, Wong et al. published a review on outcomes and prognostic factors of patients with recurrent gliomas treated within phase II controlled trials; taken the results from the 8 studies together, the progression-free survival at 6 months was 21%, median progression-free survival 10 months, and median overall survival 30 weeks [11]. These data can be considered base-line data for comparison with subsequent studies in patients with recurrent gliomas. A major brake-through was the oral alkylating agent temozolomide (TMZ) [12,13]. A multicenter phase II study of the Temodal Brain Tumor Group achieved a progression-free survival rate of 46% at 6 months; median progression-free survival was 5.4 months, and overall survival 13.6 months [14]. Alternative dosing-schedules of TMZ, such as the one-week-on/on-week off schedule showed promising results in the past [15]; only recently, results from a phase II study were released with a progression-free rate of 43% at 6 months [16]; median progression-free survival was 24 weeks, and median overall survival 38 weeks.

RT offers a local, non-systemic treatment alternative that should be considered at the time point of recurrence in patients with gliomas. Some alternatives require neurosurgical intervention, such as interstitial brachytherapy, being associated with the feared side-effects and risks of surgery. Other options offer the benefit of a non-invasive treatment, such as conventional external beam RT and high-precision RT techniques.

In the following an overview of RT choices for recurrent gliomas will be given, with description of the technical background, treatment results and ideas for future treatment concepts.

### Conventional external beam RT

Conventional RT has been used in the past to treat patients with recurrent gliomas. Small patient numbers treated with a second course of RT were published, however, the main effects were seen in at least temporary palliation [17-19]. It has been associated with high rates of side effects, whereas the clinical outcome was not convincing. Baumann et al. treated 34 patients with recurrent brain tumors with an average of 30 Gy in 3 Gy single fractions as re-irradiation [1]. Of this group, patients with recurrent GBM showed survival from re-irradiation of 2.8 months only, and patients with recurrent LGG 8.5 months, respectively. Veninga et al. published results on 42 patients receiving re-irradiation for recurrent primary brain tumors; the interval between the first and second course of RT was at least 1 year in every patient [20]. RT was deliverd with two opposing lateral fields or two wedged fields in orthogonal direction, and a median re-irradiation dose of 46 Gy (range, 4–55 Gy) in a median fractionation of 5 × 2 Gy/week was applied. The median survival and progression-free survival time after re-irradiation were 10.9 months and 8.6 months, respectively. Long-term severe complications were observed only in patients receiving more than 204 Gy cumulative biological equivalent dose (BED).

### Precision radiotherapy

Over recent years conventional RT was improved and three-dimensionally planned RT was introduced (3D-CRT). Therefore, improved target definition using CT and MRT, as well as using biological parameters such as PET and SPECT, helped improve target.

Stereotactic target localization methods with highest precision (*high precision radiotherapy*) offer optimal sparing of surrounding normal tissue. The principle of stereotactic RT was developed in the 1950s and 1960s, when Lars Leksell developed the so called Gamma Knife for the *Stereotactic Radiosurgery (SRS) *of brain tumors [21-23]. In the 1980s linear accelerators (LINAC) were also equipped for stereotactic radiation treatments. Stereotactic irradiation has been implemented widely in the clinical routine and has been proven to be effective for brain metastases, meningioma, acoustic neuroma and primary brain tumors [24-26]. The required dose can be applied in a single fraction as *Stereotactic Radiosurgery (SRS)*, as or the total dose can be applied in a number of fractions, as *Fractionated Stereotactic Radiotherapy (FSRT)*. Both modalities have been used effectively for the treatment of recurrent gliomas.

### Stereotactic radiosurgery (SRS)

SRS is a highly conformal, non-invasive, precision radiotherapy technique. Due to its accuracy and steep dose gradients at the field it is possible to deliver a prescribed dose to a defined target volume while sparing surrounding healthy tissue, including organs at risk [25,27,28].

Commonly, SRS is limited to smaller treatment volumes to prevent high incidences of radiation-induced injury; it is known that the risk of treatment-related side effects increases with target size as well as an increase in RT dose.

Shrieve et al. observed median survival times of 10.2 months after SRS for recurrent GBM; significant prognostic factors included tumor size and age [29]. At the Department of Radiation Oncology in Heidelberg, Germany, we treated 32 patients with recurrent glioma with SRS; a median dose of 15 Gy was applied to a median target volume of 10 ml. During follow up, no severe treatment related side effects could be observed, and the median survival from SRS was 10 months [27]. However, other groups have reported higher incidences of treatment related side effects, especially in larger target volumes. In a study published by Hall et al., 14% of the patients developed radiation-induced necrosis, while survival calculated from SRS was 8 months [30]. The comparably high rate of severe side-effects might be due to the relatively larger volumes (median 28 cm^3^) treated with SRS. However, the higher rate of necrosis in the study published by Hall et al. might also be due to the higher median dose of 20 Gy applied. The University of Minnesota, Department of Radiation Oncology published results of SRS in 46 patients with recurrent gliomas; a median dose of 17 Gy was prescribed to a median target volume of 30 ml; 14 out of 46 (30%) patients developed severe late complications such as necrosis [31].

A selection of reports on SRS in recurrent gliomas can be found in table 1. Data conclude that SRS is a safe and feasible treatment alternative, however, due to the higher risk of severe treatment-related side effects with increasing target size, SRS should be reserved for smaller lesions.

**Table 1:** Series of patients with recurrent gliomas treated with stereotactc radiosurgery (SRS).

**Author**	**Pt. Number**	**Histology**	**Median Dose (Gy)**	**Tumor size (ml; median)**	**Median survival (months)**	**Rate of Severe Toxiticy/Reoperation rate (%)**
Chamberlain et al, 1994	20	5 GBM, 10 AA, 5 other	13.4	17	8	-
Cho et al., 1999	46	27 GBM/19 AA	17	10	11	22%
Combs et al., 2005	32	GBM	15	10	10	-
Hall et al., 1995	35	26 GBM, 9 AA	20	28	8	31%
Kondziolka et al., 1997	23	AA	15.6	6	31	23%
Kondziolka et al., 1997	19	GBM	15	6.5	30	19%
Shrieve et al., 1995	86	GBM	13	10.1	10.2	22%

#### Fractionated stereotactic radiotherapy (FSRT)

FSRT is another non-invasive precision RT technique: The required therapeutic dose is divided into a number of fractions. By exploiting the radiobiological advantage of fractionation, the risk of side effects to normal tissue can be minimized. FSRT can be applied safely for very small target volumes as an alternative to SRS; moreover, for large tumors, FSRT can also be performed safely and effectively without the high risk of side effects associated with SRS in such tumors.

A number of groups have studied FSRT in patiens with recurrent gliomas (table 3). A very large series of patients treated at the Department of Radiation Oncology at the University of Heidelberg consisted of 172 patients treated with FSRT for recurrent gliomas [32]. In this group, median overall survival of 21 months, 50 months and 111 months could be observed for GBM, anaplastic astrocytoma or low-grade glioma recurrences, respectively. The rate of severe radiation induced side effects was very low.

**Table 3:** Series of patients with recurrent gliomas treated with hypofractionated stereotactic radiotherapy (H-FSRT).

**Author**	**Pt. Number**	**Histology**	**Tumor size (ml; median)**	**Median Dose (Gy)**	**Median Fraction Size (Gy)**	**Median survival (months)**	**Rate of Severe Toxiticy/Reoperation rate (%)**
Ernst-Stecken et al., 2006	15	GBM	22.4	35	7	-	0%
Hudes et al., 1999	19 (1)	GBM (AA)	12.6	30	3	10.5	0%
Laing et al., 1993	22	GBM	-	30–50 (range)	5–6 (range)	-	-
Selch et al., 2000	15 (3;3)	GBM (AA/LGG)	12	25	4–6 (range)	6.7	0%
Shepherd et al., 1997	29 (7)	GBM/AA (LGG)	24	20–50 (range)	5	11 (GBM/AA)	36%
Vordermark et al., 2005	10 (19)	II or III	15	30	5	13.5	26%
Vordermark et al., 2005	9 (19)	IV	15	30	5	7.4	

Other series using FSRT for recurrent gliomas are summarized in table 2. Hudes and colleagues reported a median survival of 10.5 months in a series of 20 patients treated with FSRT, with no radiation-induced late toxicity [33]. 45% of the patients improved with respect to neurological symptoms; however, tumor volumes were comparably small with 12.7 ml.

**Table 2:** Series of patients with recurrent gliomas treated with fractionated stereotactic radiotherapy (FSRT).

**Author**	**Pt. Number**	***Histology***	**Tumor size (ml; median)**	**Median Dose (Gy)**	**Median Fraction Size (Gy)**	**Median survival (months)**	**Rate of Severe Toxiticy/Reoperation rate (%)**
Cho et al., 1999	15 (10)	GBM (AA)	74	37.5	2.5	11	12%
Combs et al., 2005	71	LGG	49.3	36	2	111	-
Combs et al., 2005	42	AA		36	2	50	
Combs et al., 2005	59	GBM		36	2	21	

#### Hypofractionated stereotactic radiotherapy (H-FSRT)

Hypofractionated stereotactic radiotherapy (H-FSRT) is characterized by potentially lower toxicity than SRS, however, compared to FSRT, the risk for side effects seems increased due to the higher single doses (table 3). A major advantage of the fewer treatment fractions is the reduction of overall treatment time which is an especially important issue in terminally ill patients. Shepherd et al. treated 36 patients with H-FSRT with total doses between 20 and 50 Gy, in single fractions of 5 Gy. A major predictor of radiation damage was a total dose > 40 Gy; 36% of the patients developed steroid-dependent toxicity, and 6% of the patients required re-operation [34]. A dose escalation study published by Hudes et al. applied doses from 24 Gy to 35 Gy in median fractions of 3 Gy to 20 patients; no grade 3 toxicities could be documented, and no reoperation due to toxicity was required. Median survival from H-FSRT was 10.5 months.

A recent study published by Vordermark et al. reports on 19 patients with recurrent gliomas treated with H-FSRT [35]. The median survival from H-FSRT was 9.3 months, with 16% survival at 2 years, and a very low rate of side-effects. The strongest predictors for survival were total dose (< 30 Gy vs. 30 Gy) as well as tumor histology.

**Figure 1:**
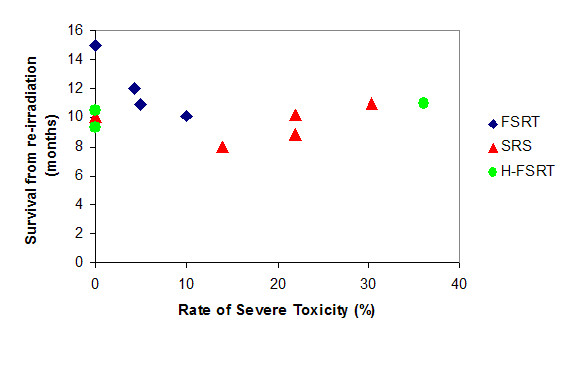
Severe treatment-related toxicity in patients with recurrent gliomas treated with FSRT, SRS of hypofractionated RT.

For each patient, the choice, whether SRS, H-FRST or FSRT is the appropriate treatment, has to be made individually, depending on the size of the lesion, the location and the previously applied RT dose and target volume. In general, it should be considered that increasing fraction size can be associated with an increase in treatment-related side effects (Fig. 1). Besides lesion size, there is a response-relationship the prescribed dose and the risk for severe treatment-related side effects [36]. Flickinger et al. proposed a model to estimate complication risk for linear accelerator radiosurgery using an integrated logistic formula [37]: Dose-volume isoeffect curves were calculated for a 3% risk of brain necrosis. The previous analysis of Kjellberg however, calculated a risk of 1% for a lesion with a diameter of 20 mm and a prescibed dose of 24 Gy. Additionally, with respect to the clinical situation, it is often difficult to distinguish between tumor progression and necrosis; moreover, is must be kept in mind that, according to the initial definition of SRS postulated by Leksell in 1951, is the aim to induce a circumsribed necrosis. Therefore, necrosis is per se not a complication of SRS, but a therapeutic effect. Therefore, the crucial aspect is that no healthy tissue should be destroyed by SRS, potentially leading to severe clinical symptoms. Several studies report rates of necrosis and/or reopreation rates, however, not in all cases can be determined whether reoperation was due to tumor progression, or mainly due to symptomatic radiation-induced necrosis.

Thus, for each clinical situation the choice whether SRS should be applied, with the advantage of short treatment times, however, with a higher risk of side effects, or whether FSRT of H-FSRT is chosen, with a lower toxicity-risk but with a longer treatment course, should be weighed against each other, taking into account such factors as patients' performance status, size and location of the lesion as well as previous therapies. H-FSRT with fewer large fractions as compared to FSRT may shorten overall treatment time, but may compromise too much of the potential advantages of fractionation.

### Intensity modulated radiotherapy (IMRT)

IMRT is another modern high-precision RT technique that offers advantages for patients with complex tumor or target volumes such as skull base or paraspinal tumors, in close vicinity to organs at risk. In such cases, better dose conformality and sparing of normal tissue can be achieved as compared to conventional conformal RT [38-40]. A major down side is considered to be an increased dose inhomogeneity within the target volume, and in some cases, an increased low dose bath to surrounding healthy tissue. Therefore, the use of IMRT for the irradiation of gliomas cannot be considered to be superior to FSRT, and the increased preparation time needed may not lead to an improved patient treatment. However, a number of groups have studied IMRT in gliomas [41-43]. Voynov et al. treated 10 patients with recurrent gliomas with IMRT in a hypofractionated regimen [43]: In 5 Gy single doses, median total doses of 30 Gy were delivered. After re-irradiation with IMRT, the median survival time was 10.1 months.

### Interstitial radiotherapy

Interstitial RT using radioactive seeds as permanent or temporary brachytherapy was performed in the 1980s in a large number of patients using high-activity iodine-125 (^125^I) or iridium-192 (^192^I; table 4).

**Table 4:** Series of patients with recurrent gliomas treated with I-125 seed implants.

**Author**	**Pt. Number**	**Histology**	**(Median) Dose (Gy)**	**Median survival (months)**	**Rate of Severe Toxiticy/Reoperation rate (%)**
***Permanent Brachytherapy***					
Gaspar et al., 1999	37 (22)	GBM (AA)	100	10.5	44%
Halligan et al, 1996	22(4)	GBM (AA)	-	16	5%
Larson et al., 2004	38	GBM	150–500	12.0	10%
Patel ell., 2000	40	GBM	120–160	11.8	0%
***Temporary Brachytherapy***					
Gutin et al., 1987	18	GBM	-	13	41%
Leibel et al., 1989	45	GBM	70	12.5	49%
Leibel et al., 1989	50	AA	70	18.7	
Shrieve et al., 1995	32	GBM	50	11.5	44%
Simon et al., 2002	42	GBM	40–60	12.5	24%
Sneed et al, 1997	45	AA	64	12.3	53%
Sneed et al., 1997	66	GBM	64	11.7	46%
***Glia Site Temporary Brachytherapy***					
Chan et al., 2005	24	GBM	53	9.1	33%
Gabayan et al., 2006	80	GBM	60 Gy	8.9	2%
Gabayan et al., 2006	15	Non-GBM	60 Gy	10.9	

Implantation of ^125^I seeds for recurrent glioma has been shown to offer excellent palliation in a majority of patients and a significant level of long-term survival. ^125^-I seeds can be used for permanent or for temporary implants. In a number of cases improved survival was seen, however, along with high rates of radionecrosis [44-47]. Seed implants can produce inhomogeneous radiation dose distributions, associated with repeated operations for radionecrosis occurring in up to 64% of patients treated with interstitial implants [47-50]. Stereotactic placement of multiple sources around a surgical cavity is a technically challenging procedure, most likely explaining the frequently occuring area of inadequate dosing; suboptimal dosimenty could explain the relatively poor results observed in some trials [46,51]. The use of low-dose-rate (LDR) interstitial brachytherapy with permanent ^125^I implants has reduced the rate of severe complications including syptomatic radionecrosis. Permanent LDR brachytherapy does not require stereotactic frame placement or the drilling of multiple holes in the skull for seed placement. However, as with high-dose-rate (HDR)-temporary implants optimal dosimetry may be difficult to achieve. Survival in patients with recurrent GBM treated with this technique is comparable to that observed in similar patients treated with brachytherapy using temporary high-acitivity implants [52,53].

Permanent brachytherapy is performed immediately after neurosurgical resetion, using ^125^I sources placed around the surgical cavity and implanted into the surrounding brain tissue. The total dose rate over the lifetime of the radiation sources lies between 100 and 400 Gy, however, the dose rate is much lower than for temporary brachytherapy, during which 50 to 65 Gy are applied over 4 to 12 days. For GBM, median survival times after permanent brachytherapy range from 10.5 months to 12 months [52-54]. Patel et al. treated 40 patients with recurrent GBM with permanent interstitial implants, with a dose between 120 and 160 Gy; none of the patients developed severe side effects such as radiation necorsis or injury, and survival after implantation was 47 weeks [53].

Using temporary brachytherapy, the groups of Sneed (n = 66 GBM, n = 45 AA), Shrieve (n = 32 GBM) and Chan (n = 24 GBM) could obtain median survival times of 11.7, 11.5, 12.3 and 9.1 months, respectively [29,55,56]. Median doses between 50 and 64 Gy were applied, and reoperation rates after treatments were betwwen 8% and 53 %. Leibel et al. reported on a series of 95 patients with recurrent malignant gliomas treated with brachytherapy after initial conventional RT (dose range 40–72 Gy); for a selected group of patients, interstitial implantation of ^125^I sources (dose range, 52.7 – 150 Gy) resulted in a median survival of 18.7 and 12.5 months for anaplastic astrocytomas and GBM, respectively [47]. In 49% of the patients re-operation was required due to treatment-associated necrosis within the high-dose area of the implantation. Gutin et al. compared the survival of 18 patients treated with ^125^I implants for recurrent GBM with 42 historical controls treated with chemotherapy. Median survival in the ^125^I-group was 52 weeks as compared to 28 weeks after chemotherapy [57].

^192^Ir-wires applied through Nylon catheters placed into the tumor recurrence under stereotactic conditions were used for temporary brachtherapy by Simon et al. [58]. 42 patients with recurrent GBM were treated with tumor doses between 15 and 60 Gy, over a 7 to 12 day period. Thereafter, radioactive wires were removed at bedside and the catheters were taken out by the neurosurgeons. Overall survival was 80% at 6 months, 48% at 1 year and 11% at 2 years; 14% of the patients experienced complications from brachytherapy, including skin necrosis.

A novel alternative temporary brachtherapy technique is intracavitary low-dose rate (LDR) brachytherapy, also referred to as Glia-site brachytherapy [59]. It is performed with an expandable balloon catheter (GliaSite Radiation Therapy System (RTS), Cytyc Surgical Products, Palo Alto, CA, USA) which is placed into the resection cavity at the time of tumor resection or debulking; the GliaSite system offers balloons with 2,3 and 4 cm diameter. After a period of 2 to 4 weeks after surgery the catheter is filled with an aqueous solution of organically bound ^125^I (Iotrex [sodium 3-(^125^I)-iodo-4-hydroxybenzenesulfonate]) for a predetermined amount of time of about 3 to 6 days until the calculated dwell time has been achieved. Thereafter, the Iotrex is retrieved transcutaneously. The feasibility and saftey of the system was published by Tatter et al.; the implantation, radiation delivery as well as the expantation were carried out without any serious side effects [59]. An initial report on the system was published by Shrieve et al. in 1995 [60].

The target volume for GliaSite brachytherapy is considered to be the residual enhancing tissue identified in postoperative MRI-scans, and it is calculated to receive at least 100% of the prescribed dose. GliaSite functions as a single spherical source of low-dose rate radiation and the photon energy of ^125^I (27–35 keV photons) result in rapid attenuation over short distances via the photoelectric effect, which leads to a steep dose fall-off and a typical prescription depth of 0.5 to 2 cm.

Gabayan et al. published a retrospective analysis of 95 patients with recurrent WHO grade III and IV gliomas treated with GliaSite [61]. The median dose applied was 60 Gy using ballon sizes between 2.0 and 4.0 cm and a median survival time calculated from re-operation was 36.3 weeks. Little information is given on the resection status and its impact on outcome after GliaSite brachytherapy; patients undergoing total or at least subtotal resection are known to present with more favourable outcomes, and no data is provided on the number of patients that were treated a biopsy only. Therefore, no conclusion can be made as to which patients profit most from GliaSite brachytherapy.

Chan et al. treated 24 patients with Glia Site brachytherapy, with a rate of 8% of symptomatic necrosis and survival times of 9.1 months [56].

### Intraoperative electron radiotherapy (IOERT)

IOERT is implemented in clinical routine expecially for rectal carcinoma, sarcomas or for pancreatic carcinomas [62,63]. Only a limited number of centers are equipped with dedicated IOERT machines. Irradiation is performed in the operating room, and RT is applied directly into the resection cavity.

Few data exist on IOERT in patients with gliomas. However, the rational of local dose escalation by an electron boost directly into the surgical cavity, especially for unresectable or only partially resectable tumors, seems a promising treatment alternative.

First promising results on IOERT were published by the groups of Sakai and Matsutani in patients with primary malignant brain tumors, however, could not be reproduced by European studies so far [64-66]. Therefore, for primary gliomas, addition of IOERT to conventional treatment (surgery and RT) has not proven to significantly improve outcome [67,68].

However, for recurrent gliomas, a number of studies have shown that IOERT is safe and feasible with acceptable results without signficantly increasing sugery times. A major downside is the "invasive" nature, i.e. IOERT can only be performed in the neurosurgical setting.

Japanese data reported by Hara et al. on 6 patients with recurrent gliomas showed superior results in patients treated with surgery and IOERT than surgery alone or in combination with chemotherapy [69]. Shibamoto et al. evaluated the feasibility of IOERT in 19 patients with recurrent brain tumors of different histologies [70]. RT had been part of the initial treatment with a mean dose of 53 Gy (range, 17–65 Gy). IOERT was applied in a dose range of 23–40 Gy. During follow up, three cases of symptomatic brain necrosis occurred, one of which was fatal. For the subgroup of patients with anaplastic astrocytomas or GBM, median survival from IOERT was 12 months.

Ortiz de Urbina published results on IOERT in 9 patients with recurrent gliomas; the actuarial 18 months survival rate and survival time was 47% and 13 months in this group. The median time to tumor progresseion after IOERT was 11 months. IOERT was applied in single doses of 10–20 Gy, and no IOERT related mortality was observed [71].

At the University Hospital of Muenster in Germany IORT has been performed since 1992 for gliomas. In total 71 patients were treated, of which 19 were treated for recurrent gliomas [67]. In these patients, electron-beam IORT was performed with 15–25 Gy, depending on size of the lesion, doses and field location of previously performed RT as well as the RT dose applied prior to IORT. Disease-free survival in this subgroup was 12.45 months. Perioperative complications were not increased in the IORT- group, however, no clear statement could be made on the incidence of severe side-effects such as brain necrosis.

### Radio-chemotherapy

To further optimize treatment results obtained by re-irradiation, chemotherapy might be added to RT. However, this combined approach is likely to increase side effects, especially in substances with strong radiosensitizing potential. As re-irradiation alone is applied with caution, radio-chemotherapy as re-irradiation is performed even more restricted.

Only few groups have studied combined re-iradiation and chemotherapy in recurrent gliomas (table 5).

**Table 5:** Series of patients with recurrent gliomas treated with radio-chemotherapy.

**Author**	**RT-Technique**	**Chemotherapy**	**Pt. Number**	**Histology**	**Tumor size (ml; median)**	**Median Dose (Gy)**	**Median Fraction Size (Gy)**	**Median survival (months)**	**Rate of Severe Toxiticy/Reoperation rate (%)**
Arcicasa et al., 1999	CH-EBRT	CCNU	24 (7)	GBM (AA)	-	34.5	1.5	13.7	-
Glass et al., 1997	CH-HSFRT	Cisplatin	13 (7)	GBM (AA)	14	35–42 (range)	3.5–6 (range)	13.7	-
Lederman et al., 2000	CH-HSFRT	Paclitaxel	88	GBM	32.7	18–36 (range)	4–9 (range)	7	11%
Wurm et al., 2006	CH-HSFRT	Topotecan	5 (25)	III	16.5	30	5	21.3	-
Wurm et al., 2006	CH-HSFRT	Topotecan	20 (25)	IV	16.5	30	5	7.9	

Wurm et al. combined HFSRT with topotecan chemotherapy in 25 patients; histology was AA in 20% and GBM in 80%, respectively. RT was applied in a median dose of 30 Gy in 5–6 fractions [72]. Overall survival was 14.5 months, 12% of the patients developed adverse RT-induces side effects. Topotecan was prescribed in a dose of 1.1 mg/m^2 ^qd as continuous infusion during H-FSRT followed by up to 48 courses of chemotherapy.

Glass et al. combined HFSRT with cisplatin weekly (40 mg/m^2^) in 20 patients with recurrent AA and GBM [73]. A median survival of 12.7 months was observed, with a rate of 15% of necrosis at re-operation. Another group combined HFSRT with paclitaxel weekly (120 g/m^2^) in 88 patients with GBM; median overall survival was 7 months [74]. Only 8% presented with necrosis at re-operation.

Thirty-one patients with recurrent GBM and AA were treated with re-irradiation and lomustine (CCNU) at the Department of Radiation Oncology in Aviano, Italy [75]. Oral administration was begun concomitantly with re-irradiation and was repeated every 6 weeks. Toxicity was moderate, however, also treatment results were observed with only modest subjective and objective response rates; on the other hand, survival from relapse was remarkable at 13.7 months.

Chamberlain et al. combined ^125^I-brachytherapy with concurrent cisplatin; in 16 patients stereotactically placed catheters were afterloaded with ^125^I sources with a median 50 Gy minimum treatment volume dose during a 100 hour period along with cisplatin 20 mg/m^2 ^qd over 5 days. Early complications included headache, seizures and worsening of neurological symptoms; late complications included radiation-induced necrosis in 9 patients requiring reoperation [76]. A partial response was seen in 5 patients, stable disease in 7 and progressive disease in 3 patients over a median follow-up time of 9.5 months.

### Hyperthermia

It is known that an increase in temperature to a certain level enhances the anti-tumor action of chemotherapy and radiotherapy, alike [77,78]. For therapeutic hyperthermia, temperatures between 44°C – 46°C are considered to be effective. Above this temperature, coagulation necrosis occurs, which is a principle used in thermo- or radio-frequency-ablation. In oncological concepts, the direct cytotoxic effect of hyperthermia is anticipated, but also the chemo- and radiation-sensitizing effect of higher temperature.

Therefore, there was hope that hyperthermia might help improve outcome in patients with gliomas. Sneed et al. recruited 112 patients within 5 years with tumor manifestations not exceeding 5 cm in diameter [79]. Within this two armed study, patients in both arms were treated with brachtherapy; in one arm, 30 min prior to brachtherapy hyperthermia was applied. Progression-free survival could be increased from 33 to 49 weeks in the group treated with hyperthermia, and overall survival could be extended 9 weeks by application of hyperthermia; 2-year survival rates were 0% and 10% for the two groups, respectively. However, technical setup and treatment procedures were complex.

### Proton radiotherapy/Carbon ion radiotherapy

Particle therapy such as proton and carbon ion treatment has been implemented for patient treatment over the last years. In certain tumor entities carbon ion RT has been shown to offer significantly better tumor control as compared to photon RT [80,81]. Protons and carbon ions as well are characterized by a distinct physical so called inverse dose profile; this results in a high dose deposition very locally in the so-called Bragg Peak, and a steep dose fall-off thereafter. Additionally, carbon ion beams offer an increased relative biological effectiveness (RBE) which is known to be especially beneficial in radioresistant, hypoxic and/or slow-growing tumors.

Worldwide, a number of centers offer particle beams; at the University of Heidelberg, carbon ion radiotherapy is performed at the Gesellschaft für Schwerionenforschung (GSI) in Darmstadt. In 2007, the Heidelberg Ion Therapy Center will take up patient treatment offering proton and carbon ion RT. Preliminary data from Japan have shown effectivity of C_12 _in patients with GBM, however, in this series, no concomitant chemotherapy was applied which is considered the standard for the treatment of primary GBM [82]. Since further investigation of C12 in patients with gliomas is warranted, implementation of C_12 _to recurrent astrocytomas will be evaluated in a clinical setting at the Department of Radiation Oncology in Heidelberg, Germany, in the near future.

## Conclusion

In the past, a number of attempts have been made to salvage patients with recurrent gliomas with a second course of radiotherapy. A number of invasive and non-invasive techniques are available and have proven effectivity in recurrent gliomas. Certainly, every patient confronts us with an individual setting, including tumor size, location, previous treatments as well as performance status and clinical symptoms. The choice as to which modality should be applied has to be made individually for each patient, reflecting possibilities, potential benefit and side-effects.

For all RT modalities, close vicinity to sensitive risk structures is a main obstacle for a second course of RT since the risk for severe side effects, confining quality of life or jeopardizing even vital organ functions, are high due to the limited tolerance dose [83]. Especially for SRS as well as invasive procedures such as brachytherapy, the potential toxicity becomes of major concern if the tumor becomes larger or is located close to eloquent structures, such as the optic pathway, basal ganglia, motor or speech areas or the brain stem.

Using modern highly conformal RT techniques, precise dose application to a defined target volume is possible while the surrounding normal tissue can be spared, in a non-invase approach. Therefore, treatment related toxicity can be minimized, while treatment results can be improved. Re-irradiation using high precision radiotherapy offers significant benefit, at least for a subgroup of patients. For each patient, the fractionation scheme must be chosen individually.

However, it is not a curative treatment and further improvement is needed urgently. Further investigation of combined radiochemotherapy as well as novel RT modalities such as carbon ion RT are needed.

## Competing interests

The author(s) declare that they have no competing interests.

## Authors' contributions

SEC and DSE collected the data useful for the analysis; SEC and DSE performed the analysis and wrote the manuscript. JD revised the article critically for important intellectual content. All authors read and approved the final manuscript.

## Pre-publication history

The pre-publication history for this paper can be accessed here:


